# Hemodynamic changes caused by acupuncture in healthy volunteers: a prospective, single-arm exploratory clinical study

**DOI:** 10.1186/s12906-017-1787-z

**Published:** 2017-05-22

**Authors:** Tae-Hun Kim, Boncho Ku, Jang-Han Bae, Jae-Young Shin, Min-Ho Jun, Jung Won Kang, Junghwan Kim, Jun-Hwan Lee, Jaeuk U. Kim

**Affiliations:** 1 Korean Medicine Clinical Trial Center, Kyung Hee University Korean Medicine Hospital, #23 Kyungheedae-ro, Dongdaemun-gu, Seoul, 02447 South Korea; 2 KM Fundamental Research Division, Korea Institute of Oriental Medicine, Daejeon, 34054 South Korea; 3 Clinical Research Division, Korea Institute of Oriental Medicine, Daejeon, 34054 South Korea; 4 Department of Acupuncture & Moxibustion, College of Korean Medicine, Kyung Hee University, Seoul, 02447 South Korea

**Keywords:** Acupuncture, Radial artery pressure pulse wave, Radial artery ultrasonography, KIOM-PAS, Hemodynamic variables

## Abstract

**Background:**

Radial pressure pulse wave (RPPW) examination has been a key diagnostic component of traditional Chinese medicine. The objective of this study was to investigate the changes in RPPW along with various hemodynamic variables after acupuncture stimulation and to examine the validity of pulse diagnosis as a modern diagnostic tool.

**Methods:**

We conducted acupuncture stimulation at both ST36 acupuncture points in 25 healthy volunteers. We simultaneously assessed the RPPW by pulse tonometry; heart rate variability (HRV) by electrocardiogram; photoplethysmogram (PPG) signals, respiration rate, peripheral blood flow velocity and arterial depth by ultrasonography; and cardiac output by impedance cardiography, before, during and after a session of acupuncture stimulation.

**Results:**

We observed consistent patterns of increased spectral energy at low frequency (<10 Hz) and pulse power using RPPW examination and in the amplitude and systolic area of the PPG signal during the entire acupuncture session. The low- and high-frequency domains of HRV increased and decreased, respectively, during the acupuncture session. The peripheral blood velocity rose shortly after needle insertion, reached a maximum in the middle of the session and decreased afterwards. The augmentation index (AIX) and pulse transit time (PTT) obtained from RPPW did not change significantly.

**Conclusion:**

Acupuncture stimulation at ST36 in healthy subjects increased the peripheral pulse amplitudes (pressure pulse wave (PPW) and PPG), blood flow velocity (ultrasonography) and sympathetic nerve activity (HRV). The lack of changes in the AIX and PTT suggests that the increased pulse amplitudes and blood flow velocity may result from increased cardiac output.

**Trial registration:**

Clinical Research Information Service (KCT0001663).

**Electronic supplementary material:**

The online version of this article (doi:10.1186/s12906-017-1787-z) contains supplementary material, which is available to authorized users.

## Background

Pulse diagnosis is a specific diagnostic and prognostic method used in traditional East Asian medicine (TEAM), including traditional Chinese medicine, traditional Korean medicine, and traditional Kampo medicine. Examining the pressure pulse wave (PPW) along arteries, especially along the radial artery, using the index, middle and ring fingers is a common practice in TEAM clinics [1]. Pulse diagnosis is a key to understanding TEAM medical theory and practice. Although there have been marked quantitative and qualitative improvements in the development of scientific imaging devices and laboratory tests, pulse diagnosis has occupied an important position in TEAM diagnosis. However, despite its clinical importance, very little is known about the physiological and pathological mechanisms underlying pulse diagnosis.

From the viewpoint of modern medicine and biological science, the pulse is understood as a quantity that primarily reflects cardiac and hemodynamic activities [2]. The PPW is a wave that propagates along arteries and originates from the periodic blood pumping of the heart and vasodilation: the PPW travels approximately 15 times faster than the flow of the blood in the aorta. The PPW travels from the aortic arch to the peripheral arteries and carries vital information, such as heartbeat, blood pressure, arterial stiffness and aging [2]. These vital signs are affected by various body conditions. For instance, arterial endothelial function [3], the severity of arteriosclerosis [4], obesity [5] and sympathetic tone [6] have been identified as significant factors affecting PPW changes. Nevertheless, many research groups have attempted to reveal more complex factors related to PPW that can reflect health status either directly or indirectly.

Revealing the connection between the behavior of the PPW and various body conditions is a way to modernize and revitalize the pulse diagnosis of TEAM. Investigating the relationship between PPW and other cardiac and hemodynamic bio-signals may elucidate the underlying mechanism of pulse diagnosis and will help promote it as a modern scientific diagnostic method. In this sense, studying the continuous and simultaneous assessments of various hemodynamic factors associated with PPW changes is an important research agenda for understanding and modernizing the pulse diagnosis of TEAM.

A popular treatment modality that affects the characteristics of the PPW is acupuncture. According to the emperor’s inner canon (Huangdi Neijing), “The key to acupuncture treatment is Qizhi, which brings about the therapeutic effect of acupuncture,” where Qizhi may refer to the changes in the pulse wave [7]. Therefore, the effectiveness of acupuncture in a patient’s treatment should be observable by pulse diagnosis and should modify the characteristics of the pulse wave. Indeed, several studies have reported that acupuncture can affect various bio-signals, such as heart rate variability (HRV) [8] and arterial blood flow [9], as well as the PPW [10]. These studies discussed changes in only one or two hemodynamic variables in relation to the acupuncture treatment, and so far no study analyzed various bio-signals simultaneously to investigate integrative hemodynamic phenomena occurred by acupuncture treatment. The objective of this study was to assess the radial PPW (RPPW) and several other bio-signals to obtain an overall picture of the hemodynamic changes in the human body caused by acupuncture stimulation and to help provide a comprehensive understanding of the mechanism underlying the pulse diagnosis of TEAM. The present study experimentally assessed changes in various bio-signals before, during and after ST36 acupuncture stimulation. Healthy young participants attended a single acupuncture session, and bio-signals including radial artery pulse pressure, blood flow and cardiac output were assessed approximately 7 times during the acupuncture session.

## Methods

This study was a prospective, single-arm, exploratory clinical study to observe the effect of acupuncture stimulation on various hemodynamic variables, such as RPPW, in healthy young participants. This study was conducted at the Korean Medicine Clinical Trial Center, Kyung Hee University Korean Medicine Hospital, Seoul, South Korea, from October 2015 to December 2015. The study protocol was approved by the Institutional Review Board of Kyung Hee University Korean Medicine Hospital, Seoul, Korea (KOMCIRB-150818-HR-030) and was registered with the Clinical Research Information Service (registration number: KCT0001663) before the first participant was included. Written informed consent was obtained from each participant prior to study participation. Twenty-five healthy adults were recruited through advertisements on bulletin boards at the local hospital and university. Through history taking and assessment of vital signs including blood pressure, respiratory rate, pulse rate and body temperature, we evaluated general health status of the potential participants. The detailed protocol for the study is described in Shin et al. [11].

### Inclusion criteria

The following inclusion criteria were applied as follows: healthy participants aged between 20 and 30 years old who had provided signed written informed consent.

### Exclusion criteria

Participants who met the following criteria were excluded from this study:

1. Use of medications within 1 month before participation, including antihypertensives, hypoglycemic agents, narcotics, tranquilizers, antithrombotic or antiplatelet agents, anticoagulants, or hormone drugs
2. Pregnancy or lactation
3. Korean Medicine College students or Korean medical doctors
4. Acupuncture treatment within the last 4 months
5. Inability to undergo evaluation with the pulse tonometric device or ultrasonography
6. A history of heart disease or transplanted devices such as pacemakers
7. Participation in other clinical trials within the last 3 months
8. Communication disorder
9. Drug addiction or alcohol abuse
10. Conditions where acupuncture might not be safe, such as metal allergy
11. Refusal to participate in the trial or provide informed consent
12. Exclusion at the investigator’s discretion

### Study procedures

**Fig. 1:**
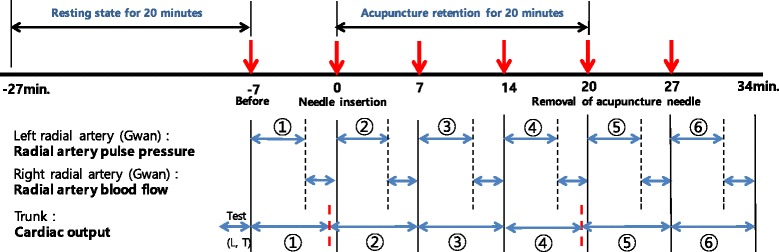
Study procedure for the assessment of hemodynamic and RPPW variables at the following points: pre-acupuncture, immediately after needle insertion, during needle insertion, and removal of needle

**Fig. 2:**
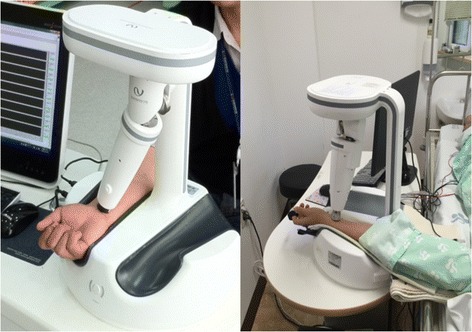
The overall features of KIOM-PAS for the assessment of radial artery pulse pressure in different positions (*left*: sitting position, *right*: lying position). The figure was epitomized from the previous article that described the study protocol of this study [11]

For preparation, the participants were not allowed to overeat, perform physical exercise or undergo emotional excitement for 1 week before the assessment, and smoking, and any caffeinated drinks such as coffee or Coke were prohibited on the assessment day. The participants who were included in this study after the screening test underwent several sessions of assessments of various bio-signals before, during and after a one-time acupuncture needling at ST36 on both legs during the procedure (Fig. 1). First, the Global Physical Activity Questionnaire (GPAQ) and the Credibility/Expectancy Questionnaire (CEQ) were completed before the assessment. After a 20-min bed rest, the spectral energy from 10 to 30 Hz (SE_10-30Hz_), spectral energy from 0 to 10 Hz (SE_0-10Hz_), pulse power index (PPI), pulse depth index (PDI), and pulse volume index (PVI) were measured on the left wrist of each subject with an appropriate pulse tonometric device (KIOM-PAS, Korea Institute of Oriental Medicine, Daejeon, Korea; Fig. 2). HRV, photoplethysmogram (PPG) signals, cardiac output (CO), and respiration (RSP) were measured using a physiological data acquisition system on the chest and nasal openings (Biopac module, Biopac MP150, Biopac Systems Inc., USA). The velocity of the blood flow and the diameter and depth of the blood vessel were measured using ultrasonography (Voluson 730 Pro, GE Healthcare Austria GmbH & Co OG, Austria) on the right wrist.

After assessing these bio-signals, the physician conducted the acupuncture procedure at both ST36 points, which were located according to the ‘WHO Standard Acupuncture Point Location in the Western Pacific Region’ [12]. A 0.16 mm × 40.0 mm disposable stainless steel acupuncture needle (Seirin Co. Ltd., Shizuoka, Japan) was used for needling. The Deqi sensation was induced through bidirectional rotation at a right angle (90°) for 18 s, and the needles were retained for 20 min before removal. Certified Korean Medicine Doctors (KMDs) with more than 10 years of clinical experience and 6 years of education performed the acupuncture treatments. All measurements were performed six times in total. After the measurements were complete, the subjects were given the Acupuncture Sensation Questionnaire (ASQ) [13], and adverse events related to the acupuncture and the measurement of each outcome were assessed.

### Outcomes

The medical history of the participants, including their current medication status, surgical history, presence of other diseases, and the results of electrocardiography and pregnancy tests were recorded at baseline. Data regarding lifestyle factors, including exercise, smoking, caffeine intake, and alcohol consumption, were documented. Hypertension, height, weight, and other demographic data were also obtained.

GPAQ is an instrument for assessing physical activity. This questionnaire collects information on physical activity participation in three settings (or domains) and on sedentary behavior using 16 questions (P1-P16). The domains are “activity at work,” “travel to and from places,” and “recreational activities” [14]. CEQ was recently developed as a measure of treatment credibility and expectancy. This questionnaire consists of 1 question that reflects the expectations for acupuncture and rates on a scale of 1–9 [15]. ASQ was developed by the Department of Applied Korean Medicine, Kyung Hee University, Seoul, Korea, for the measurement of acupuncture sensation. This questionnaire consists of 3 parts: the sensations during the insertion (SIA), manipulation (SMA), and maintenance of the acupuncture needle (SM). The questionnaire consists of 33 items for SIA, 59 for SMA, and 29 for SM, and each sensation of acupuncture is evaluated with a 10-cm visual analog scale (VAS) for every item [13]. GPAQ, CEQ and ASQ were all used after acupuncture needling.

Details of RPPW and other bio-signal parameters are presented in Table 1. To assess the RPPW, we used KIOM-PAS, a pulse tonometric device developed at the Korea Institute of Oriental Medicine; this device is used for the assessment of radial artery pulse pressure [16]. KIOM-PAS consists of a main body and a sensing body composed of a piezoresistive 7-channel sensor with the sensors arranged in a row. In this study, the operator measured the pulse signals at Guan during the procedure. SE_10-30Hz_ (the primary outcome of this study), SE_0-10Hz_, PPI, PDI, PVI, systolic area, diastolic area, subendocardial viability ratio (SEVR) and radial augmentation index (AIX) were extracted from the KIOM-PAS for every measurement.

**Table 1:** Description of parameters related with the radial artery pulse-pressure wave and other bio-signal characteristics

Variable (units)	Description
Radial pressure pulse wave	
SE_0-10Hz_ (V_rms_^2^, 10^−1^)	Sum of the spectral energy within 0–10 Hz
SE_10-30Hz_ (V_rms_^2^, 10^−3^)	Sum of the spectral energy within 10–30 Hz
PPI (V)	Pulse power index; Maximum amplitude of the voltage response in radial artery pulse
PDI (mm)	Pulse depth index; measure of the pulse depth based on the sensor displacement in the direction normal to the skin surface
Systolic area (Vs)	Area of systolic phase in average pulse
Diastolic area (Vs)	Area of diastolic phase in average pulse
SEVR (%, 10^−2^)	Subendocardial viability ratio; ratio between the diastolic and systolic area
AIX (%)	Radial augmentation index normalized to a heart rate of 75 bpm; (late systolic pressure/systolic pressure) × 100
Heart rate variability	
NN (s)	Normal to normal interval
SDNN (ms)	Standard deviation of the NN interval
RMSSD (ms)	Square root of the mean squared differences of NN intervals
TF (ms^2^)	Total frequency power within 0–0.4 Hz
LF (ms^2^)	Low frequency power within 0.04–0.15 Hz
HF (ms^2^)	High frequency power within 0.15–0.4 Hz
nLF (%)	LF power in normalized units; LF/(LF + HF)
nHF (%)	HF power in normalized units; HF/(LF + HF)
LF/HF	Ratio of low to high frequency components; LF/HF
Photoplethysmogram signals	
PPG amplitude (V)	Maximum amplitude in average PPG
PPG systolic area (Vs)	Area of systolic phase in average PPG
Respiration signals	
Respiration rate (bpm)	Number of respirations per minute.
Impedance cardiography	
SV (ml)	Stroke volume: volume of blood pumped from the left ventricle per beat
CO (ml/min)	Cardiac output: volume of blood being pumped from the heart per minute; stroke volume × heart rate
Combined variable from radial pressure pulse wave and electrocardiography	
PTT (ms)	Time delay between the R-peak of the ECG and the peak of the RPPW

Bio-signal variables, including HRV, CO, PPG and respiration rate, were assessed using a Biopac MP150 (Biopac module, Biopac Systems Inc., USA). Specifically, the electrocardiogram (ECG) signal was recorded using an ECG100C module with lead II to analyze HRV, and the impedance cardiography signal was recorded using a NICO100C module to calculate the stroke volume (SV) and CO. The PPG signal was acquired using a PPG100C module to determine the average amplitude and average systolic area, and the RSP signal was acquired using an SKT100C module to calculate the respiration rate. Finally, the pulse transit time (PTT) was calculated by analyzing the time delay between the ECG and the RPPW.

For ultrasonography, a medical ultrasound scanner (Voluson 730 Pro, GE Medical, USA) was used with a 12 L probe. The diameter of the artery was measured in the B mode, and the maximum and average of the blood flow velocity were measured in color Doppler mode. The angle of incidence for the ultrasound was maintained at 60° in the color Doppler mode and 20° in the B mode.

All unexpected responses related to the acupuncture treatment and measurements (adverse events) were reported to the investigators by the participants or were examined by an investigator. Adverse events were evaluated by investigators as mild, moderate, or severe according to the World Health Organization Draft Guidelines for Adverse Event Reporting [17] and Spilker’s criteria [18].

### Statistical analysis

All statistical analyses were performed using the current version of R statistical software [19]. The sample size was determined based on the results of a previous study performed by Huang et al. [20]. In this study, the spectral energy of 13 to 50 Hz (SE_13-50Hz_) showed a significant mean change between pre- and post-acupuncture stimulation. Based on this result, we derived a mean change of 3.38 and a common standard deviation of 5.23 using the sample size and *p*-value presented in this paper. We planned to recruit 25 participants to detect differences with a 5% type I error, 80% power and 5% dropout rate. The significance level for all tests was set to 0.05 (two-sided). The baseline characteristics of the participants were described with the available data. Categorical outcomes were represented as the numbers of participants and percentages, and continuous variables were summarized as the means and standard deviation for normal data and the medians and interquartile range (25^th^ and 75^th^ quartiles) for skewed data. The Shapiro-Wilk test was used to verify the normality of the quantitative measures. Analyses of the primary and secondary outcomes were conducted on a full analysis set on the basis of the intention-to-treat principle and on per-protocol subsamples for the purpose of sensitivity analyses. The last observation carried forward (LOCF) method was used to impute missing data.

Although the sample size in this study was obtained from SE_13-50Hz_, it had almost the same properties as those obtained from SE_10-30Hz_ [21]. Thus, the primary outcome was chosen as the change in SE_10-30Hz_ between pre- and post-acupuncture stimulation measured at the baseline and at the end, respectively.

A paired two-sample t-test was used to determine the significance of the change induced by the acupuncture stimulation. The results of the analysis were presented as the means, standard deviation, 95% confidence intervals (CI), t-values and *p*-values. The changes in SE_10-30Hz_ at six time points (before acupuncture stimulation, immediately during needle insertion, during the stimulation at 7 and 14 min after needle insertion, and immediately and 7 min after removing acupuncture stimulation) were investigated using a linear mixed-effect model (LMM) as a secondary analysis. The LMM included covariates (sex, age, BMI, pulse rate, GPAQ, CEQ and ASQ scale effects) and interaction terms between sex and time effects. The model was modified by excluding the interaction term if it was not significant. The ANOVA table for the model was provided, and the least square mean, standard error, and 95% CI were provided for each time point. Differences in a value between a given time point and baseline (before acupuncture) were examined using Dunnett’s test.

## Results

**Fig. 3:**
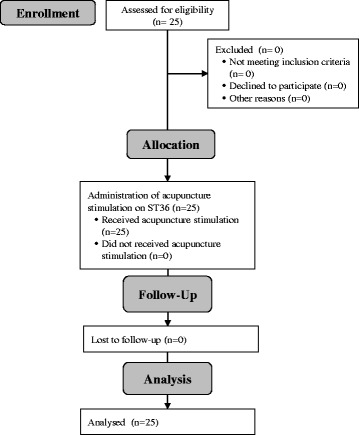
CONSORT diagram for the study. Each *rounded rectangle* with a *gray background* indicates a procedure of the study, and the rectangles with a *white background* show the number of dropout cases and the reasons

From October to December 2015, 25 healthy volunteers participated in this study, including 13 female and 12 male young adults (average age 23.3 years, standard deviation (SD) 2.4 years). No participants dropped out due to compliance problems related to acupuncture stimulation or repeated assessments (Fig. 3). Most participants had low to moderate levels of physical activity (64%). The participants were of average fitness or were slightly underweight (average body mass index (BMI) of 21.7 (2.5)). Nineteen participants had a previous acupuncture experience (76%). The expectation of the effect of acupuncture was considerably high (mean 6.5, SD 5.6, Table 2).

**Table 2:** Demographic characteristics of the participants

Characteristics	Value (Total number = 25)
Sex (n, %)	
Female	13 (52.0)
Male	12 (48.0)
Smoking (n, %)	
None	2 (8.0)
Former smoker	23 (92.0)
Caffeine (n, %)	
None	12 (48.0)
Former user	1 (4.0)
Current user	12 (48.0)
Alcohol (n, %)	
None	15 (60.0)
Former consumer	1 (4.0)
Current consumer	9 (36.0)
Level of physical activity (n, %)^a^	
Low	15 (60.0)
Moderate	1 (4.0)
High	9 (36.0)
Experience of Acupuncture (n, %)	
No	6 (24.0)
Yes	19 (76.0)
Age (mean, SD)	23.3 (2.4)
Height (mean, SD)	168.0 (8.9)
Weight (mean, SD)	61.7 (11.3)
BMI (mean, SD)	21.7 (2.5)
Systolic BP (mean, SD)	111.6 (10.3)
Diastolic BP (mean, SD)	73.6 (5.6)
Pulse (mean, SD)	74.8 (5.6)
Body temperature (mean, SD)	36.3 (0.2)
Expectation for acupuncture (mean, SD)^b^	6.5 (0.8)
Acupuncture-sensational score (mean, SD)^c^	1.5 (1.6)

^a^Physical activity level was assessed through the global physical activity questionnaire

^b^Expectation for acupuncture was assessed through the credibility/expectancy questionnaire

^c^Acupuncture sensation was assessed through the acupuncture sensation questionnaire

### Effect on the radial artery pressure pulse wave

Significant increases in SE_0-10Hz_ were observed as the acupuncture session progressed. There was no difference between baseline and immediately after needle insertion (MD 0.49, 95% CI [−0.33, 1.31]), but the differences increased continuously after 7 min (MD 0.95, 95% CI [0.13, 1.76]) and 14 min (MD 1.20, 95% CI [0.38, 2.02]), immediately after needle removal (MD 0.139, 95% CI [0.58, 2.23]) and 7 min after needle removal (MD 1.79, 95% CI [0.98, 2.61]). SE_10-30Hz_, however, did not show any differences between before and after acupuncture needling, except at the final assessment (MD −0.77, 95% CI [−1.44, −0.11]).

PPI also showed an increasing pattern in the difference between the baseline and after needling values. No significant differences were observed immediately after needling (MD 0.12, 95% CI [−0.05, 0.28]) or 7 min after needling (MD 0.12, 95% CI [−0.05, 0.28]), but the differences increased gradually after 14 min (MD 0.24, 95% CI [0.08, 0.41]), at needle removal (MD 0.27, 95% CI [0.11, 0.44]) and 7 min after needle removal (MD 0.31, 95% CI [0.15, 0.48]). The PDI values did not show significant differences for any measurement. The PVI values showed significant increases in the measurements 7 min after (MD 0.46, 95% CI [0.16, 0.71]) and 14 min after needle insertion (MD 0.32, 95% CI [0.03, 0.61]).

The systolic area and diastolic area also showed an increasing pattern during the procedure. Significant differences were observed 14 min after needle insertion (MD 54.50, 95% CI [15.50, 93.51]), at needle removal (MD 61.68, 95% CI [22.68, 100.68]), and at the final assessment (MD 92.49, 95% CI [53.48, 131.49]) for the systolic area and at needle removal (MD 12.19, 95% CI [0.03, 24.35]) and at the final assessment (MD 18.09, 95% CI [5.93, 30.25]) for the diastolic area. SEVR showed an opposite decreasing tendency after needle insertion, and significant differences were observed 14 min after needle insertion (MD −2.58, 95% CI [−5.04, −0.12]) and immediately after (MD −2.48, 95% CI [−4.94, −0.02]) and 7 min after needle removal (MD −3.35, 95% CI [−5.81, −0.89]). AIX and PTT did not show significant differences between baseline and any assessment (Table 3).

**Table 3:** Results of assessments on the variables related to the radial artery pressure-pulse wave

Variables	Assessment schedule					
	−5 min	0	+7 min	+14 min	+20 min	+27 min
	Baseline (Reference)^a^	Needle insertion^a^	During needle retention^a^		Needle removal^a^	Final^a^
SE_10-30Hz_ (10^−3^∙Vrms^2^)	1.76 (−0.39, 3.92)	−0.05 (−0.71, 0.61)	−0.45 (−1.11, 0.21)	−0.26 (−0.92, 0.40)	−0.34 (−1.00, 0.32)	−0.77* (−1.44, −0.11)
SE_0-10Hz_ (10^−1^∙Vrms^2^)	7.78 (5.37, 10.19)	0.49 (−0.33, 1.31)	0.95* (0.13, 1.76)	1.20** (0.38, 2.02)	1.39*** (0.58, 2.21)	1.79*** (0.98, 2.61)
PPI (V)	3.20 (2.68, 3.71)	0.12 (−0.05, 0.28)	0.12 (−0.05, 0.28)	0.24** (0.08, 0.41)	0.27*** (0.11, 0.44)	0.31*** (0.15, 0.48)
PDI (mm)	6.96 (4.98, 8.94)	0.08 (−0.44, 0.59)	−0.22 (−0.73, 0.29)	−0.22 (−0.73, 0.30)	−0.45 (−0.96, 0.06)	−0.34 (−0.85, 0.17)
PVI (mm)	2.51 (1.99, 3.03)	0.21 (−0.08, 0.51)	0.46*** (0.16, 0.71)	0.32* (0.03, 0.61)	0.13 (−0.16, 0.42)	0.27 (−0.03, 0.56)
Systolic area (Vs)	575.61 (490.04, 661.18)	32.09 (−6.91, 71.10)	23.72 (−15.29, 62.72)	54.50** (15.50, 93.51)	61.68*** (22.68, 100.68)	92.49*** (53.48, 131.49)
Diastolic area (Vs)	220.79 (196.43, 245.15)	3.76 (−8.40, 15.92)	2.39 (−9.77, 14.55)	10.25 (−1.91, 22.41)	12.19* (0.03, 24.35)	18.09** (5.93, 30.25)
SEVR (%)	39.33 (33.43, 45.23)	−1.58 (−4.04, 0.88)	−1.60 (−4.06, 0.86)	−2.58* (−5.04, −0.12)	−2.48* (−4.94, −0.02)	−3.35** (−5.81, −0.89)
AIX (%)	39.28 (28.40, 50.17)	1.81 (−5.98, 9.60)	0.49 (−7.30, 8.28)	−2.93 (−10.72, 4.86)	1.12 (−6.66, 8.91)	1.70 (−6.09, 9.49)
PTT (ms)	284.97 (267.79, 302.15)	0.67 (−5.18, 6.52)	3.17 (−2.69, 9.02)	1.80 (−4.05, 7.65)	4.87 (−0.99, 10.72)	4.22 (−1.64, 10.07)

*SE*_*10-30Hz*_ spectral energy of 10 to 30 Hz, *SE*_*0-10Hz*_ spectral energy of 0 to 10 Hz, *PPI* pulse power index, *PDI* pulse depth index, *PVI* pulse volume index, *SEVR* subendocardial viability ratio, *AIX* radial augmentation index, *PTT* pulse transit time

^a^Values represent adjusted mean differences between the baseline and each assessment during the needling procedure as well as their 95% CI based on the result of LMM for each parameter. Mean differences were adjusted for age, sex, height, pulse rate, BMI, GPAQ, CEQ and experience of acupuncture. Dunnett’s test was applied to control the family-wise error rate due to the multiple comparisons of the mean difference between baseline and each assessment stage for each variable. The statistical significance was indicated with asterisks: ***,*p* < 0.001; **,*p* < 0.01; *,*p* < 0.05

### Effects on bio-signal variables

For HRV, the variables showed significant differences from the baseline value only at the last measurement session. The variables were NN (MD −0.02, 95% CI [−0.05, −0.00]), HF (MD −97.81, 95% CI [−190.71, −4.90]), nLF (MD 9.50, 95% CI [1.26, 17.74]), nHF (MD −9.40, 95% CI [−17.22, −1.58]) and LF/HF (MD 0.98, 95% CI [0.02, 1.95]). Similarly, PPG systolic area showed a significant increase from the baseline only at the last measurement session (average systolic area, MD 12.00, 95% CI [2.57, 21.43]).

CO and SV, which were estimated with the impedance cardiography device, did not present any significant change between baseline and any measurement throughout the entire procedure. Lastly, respiratory rate also did not show any significant changes during the study (Table 4).

**Table 4:** Results for the bio-signal variables

Variables	Assessment schedule					
	−5 min	0	+7 min	+14 min	+20 min	+27 min
	Baseline (Reference)^a^	Needle insertion^a^	During needle retention^a^		Needle removal^a^	Final^a^
Heart rate variability (HRV)						
NN (s)	0.89 (0.81, 0.96)	0.01 (−0.02, 0.03)	−0.01 (−0.03, 0.01)	−0.01 (−0.04, 0.01)	−0.02 (−0.04, 0.01)	−0.02* (−0.05, −0.00)
SDNN (ms)	60.89 (49.54, 72.24)	2.97 (−5.84, 11.78)	0.82 (−7.98, 9.63)	3.22 (−5.59, 12.02)	1.41 (−7.40, 10.22)	4.26 (−4.54, 13.07)
RMSSD (ms)	41.51 (28.39, 54.63)	0.76 (−4.56, 6.09)	−2.21 (−7.53, 3.12)	−1.44 (−6.76, 3.88)	−3.15 (−8.47, 2.17)	−3.19 (−8.51, 2.13)
TF (ms^2^)	1407.53 (816.28, 1998.78)	−190.15 (−617.85, 237.55)	−91.18 (−518.88, 336.52)	−69.81 (−497.51, 357.89)	−13.75 (−441.45, 413.96)	26.67 (−401.03, 454.37)
LF (ms^2^)	432.39 (246.02, 618.75)	−54.68 (−203.00, 93.64)	−45.23 (−193.55, 103.09)	−45.86 (−194.18, 102.45)	−12.04 (−160.36, 136.27)	77.89 (−70.42, 226.21)
HF (ms^2^)	256.67 (25.13, 488.20)	−17.95 (−110.86, 74.96)	−42.69 (−135.60, 50.22)	−62.72 (−155.63, 30.18)	−68.12 (−161.03, 24.79)	−97.81* (−190.71, −4.90)
nLF (%)	53.03 (43.27, 62.79)	−2.90 (−11.14, 5.34)	5.01 (−3.23, 13.25)	4.26 (−3.97, 12.50)	7.10 (−1.13, 15.34)	9.50* (1.26, 17.74)
nHF (%)	43.72 (34.52, 52.93)	2.39 (−5.43, 10.21)	−5.11 (−12.92, 2.71)	−4.31 (−12.13, 3.51)	−7.23 (−15.05, 0.58)	−9.40* (−17.22, −1.58)
LF/HF	2.03 (1.08, 2.98)	−0.37 (−1.34, 0.60)	0.29 (−0.67, 1.26)	0.13 (−0.84, 1.09)	0.14 (−0.83, 1.10)	0.98* (0.02, 1.95)
Respiration						
Respiratory rate (bpm)	14.49 (10.94, 18.04)	0.11 (−1.05, 1.27)	0.43 (−0.74, 1.59)	−0.21 (−1.37, 0.96)	0.22 (−0.94, 1.38)	−0.15 (−1.31, 1.02)
PPG signals						
Amplitude (V)	0.38 (0.29, 0.47)	−0.01 (−0.05, 0.03)	0.02 (−0.02, 0.06)	0.02 (−0.02, 0.06)	0.01 (−0.03, 0.05)	0.03 (−0.01, 0.07)
Systolic area (Vs)	94.50 (71.11, 117.90)	0.18 (−9.25, 9.61)	8.17 (−1.26, 17.60)	8.73 (−0.70, 18.16)	7.26 (−2.17, 116.69)	12.00** (2.57, 21.43)
ICG signals						
Cardiac output (ml/min)	7107.78 (2142.10,12,073.47)	−182.77 (−1218.29, 852.76)	−395.31 (−1430.84, 640.21)	−509.858 (−1545.39, 525.67)	−261.268 (−1296.80, 774.26)	−239.630 (−1275.16, 795.90)
Stroke volume (ml)	105.659 (16.99, 194.32)	−5.084 (−21.76, 11.60)	−8.855 (−25.54, 7.83)	−10.239 (−26.92, 6.44)	−11.781 (−28.46, 4.90)	−11.699 (−28.38, 4.98)

*NN* normal to normal interval, *SDNN* standard deviation of the NN interval, *RMSDD* square root of the mean squared differences of NN intervals, *TF* total frequency, *LF* low-frequency domain, *HF* high-frequency domain, *nLF* normalized low-frequency domain, *nHF* normalized high-frequency domain

**,*p* < 0.01; *,*p* < 0.05

^a^Values represent differences between the baseline and each assessment during the needling procedure (95% CI). The other details are identical to the footnote of Table 3 except for following abbreviations

### Effects on the variables of radial artery ultrasonography

The diameter of the radial artery did not show significant changes throughout the entire procedure. However, the maximum blood velocity increased immediately after needle insertion (MD 2.30, 95% CI [1.46, 3.15]), reached the maximum value 7 min after needling (MD 2.46, 95% CI [1.62, 3.31]), and subsequently decreased significantly until 14 min after needling (1.35, 95% CI [0.50, 2.19]); subsequently, no differences were observed immediately after needle removal or 7 min after needle removal (0.54, 95% CI [−0.30, 1.39] and 0.19, 95% CI [−0.66, 1.03], respectively). The average blood flow velocity showed a similar pattern as the maximum velocity. Significant increases were observed immediately after needling (MD 4.09, 95% CI [2.60, 5.59]) and during needle retention (MD 4.52, 95% CI [3.02, 6.01] after 7 min and MD 2.45, 95% CI [0.96, 3.95] after 14 min), but there were no differences immediately after needle removal (1.10, 95% CI [−0.40, 2.59]) or 7 min after needle removal (0.30, 95% CI [−1.19, 1.80], Table 5).

**Table 5:** Results of assessments on the variables from radial artery ultrasonography

Variables	Assessment schedule					
	−5 min	0	+7 min	+14 min	+20 min	+27 min
	Baseline (Reference)^a^	Needle insertion^a^	During needle retention^a^		Needle removal^a^	Final^a^
Diameter of radial artery (mm)	2.01 (1.83, 2.19)	0.08 (−0.06, 0.21)	−0.00 (−0.14, 0.13)	0.06 (−0.08, 0.19)	0.00 (−0.13, 0.14)	−0.07 (−0.20, 0.07)
Maximum velocity of blood flow (cm/s)	5.29 (1.92, 8.65)	2.30*** (1.46, 3.15)	2.46*** (1.62, 3.31)	1.35** (0.50, 2.19)	0.54 (−0.30, 1.39)	0.19 (−0.66, 1.03)
Average velocity of blood flow (cm/s)	9.31 (3.50, 15.11)	4.09*** (2.60, 5.59)	4.52*** (3.02, 6.01)	2.45** (0.96, 3.95)	1.10 (−0.40, 2.59)	0.30 (−1.19, 1.80)

***,*p* < 0.001; **,*p* < 0.01;

^a^Values represent differences between the baseline and each assessment during the needling procedure (95% CI). The last details are identical to the footnote of Table 3

## Discussion

**Fig. 4:**
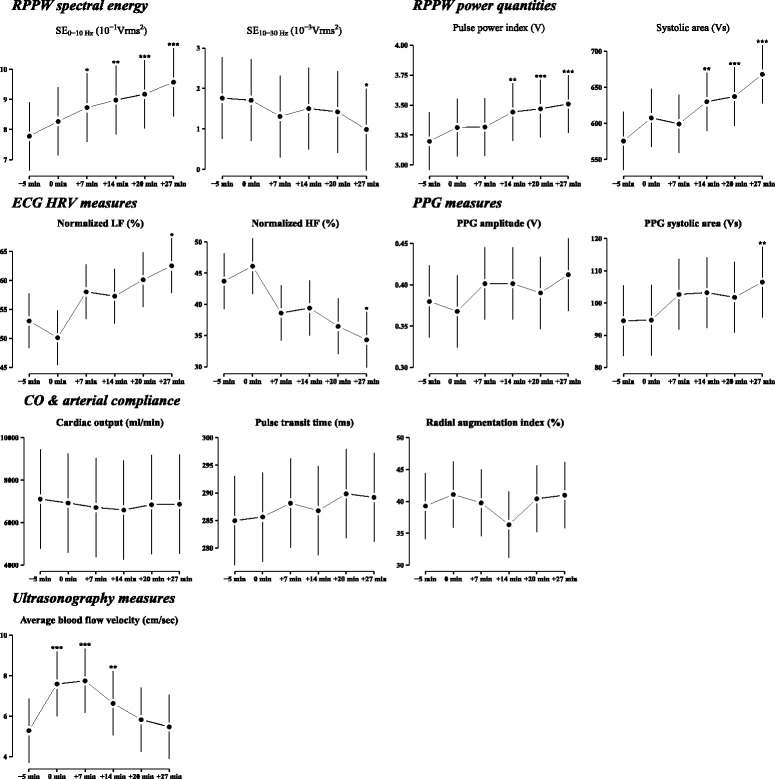
Estimated mean profiles of hemodynamic and RPPW measures for the main discussion. Each *dot* and *line* represent the least squared mean and its standard error, derived from the identical LMM used in the results shown in Tables 3, 4 and 5. *Asterisks* indicate the magnitude of statistical significance for the mean difference between baseline (−5 min) and the other assessment stages. Detailed descriptions of the tests are provided in Table 3, 4 and 5. The family-wise type I error rate was adjusted with Dunnett’s test. ***,*p* < 0.001; **,*p* < 0.01; *,*p* < 0.05

We found that RPPW and various hemodynamic variables, including peripheral blood velocity, HRV and PPG, followed consistent patterns during the acupuncture session at ST36 (Fig. 4). In the RPPW analysis, significant increases were observed in the low-frequency domain of the power spectral density (SE0-10 Hz), pulse power index (PPI), and systolic and diastolic area, while a decreasing pattern was observed for SEVR (Additional file 1: Figure S1). Likewise, the amplitude and systolic area of the PPG signal showed a continuously increasing pattern during the entire session. Peripheral blood velocity, which was assessed by ultrasonography, began to rise shortly after needle insertion, reached its maximum in the middle of the session and decreased during the rest of the retention period and after needle removal. Together, these changes indicate that the acupuncture stimulation at ST36 induced an increase in blood flow velocity (Additional file 1: Figure S3) and, consequently, pulse pressure (PPI and SE0-10 Hz). This increase was mostly driven by the enhanced systolic blood pressure (SEVR and PPG).

To investigate autonomic nerve activity, we acquired HRV data using ECG. From the ECG measurements, we found a tendency toward an increase in the low-frequency domain (0.04–0.15 Hz) of HRV and a decrease in the high-frequency domain (0.15–0.4 Hz) during the acupuncture session, although these changes did not reach statistical significance. These responses imply that the acupuncture treatment induced an increase in sympathetic activity and a decrease in parasympathetic activity. Typical physiological changes associated with increases in sympathetic tone are an increase in cardiac output (CO) and/or vascular constriction [22].

We assume that these hemodynamic changes are consequences of either increased CO and/or decreased arterial compliance. We calculated AIX and PTT from the RPPW and estimated the CO using an impedance cardiography module [23]. Previous studies have reported that acupuncture affected the blood flow in the peripheral arteries during the acupuncture session, and one possible mechanism was related to the regulation of systemic vascular resistance through the modulation of sympathetic tone without any changes in pulse rate, blood pressure or CO [9, 24]. Similarly, we found that CO did not change during the entire acupuncture session. We also found that AIX and PTT did not change during the entire acupuncture session. AIX and PTT can be used as surrogate indicators of arterial compliance (AIX and PTT) and peripheral resistance (AIX) [25]. Therefore, the lack of change in either AIX or PTT verifies that arterial compliance was not affected by the acupuncture stimulation. Hemodynamic changes, such as blood flow velocity or pulse pressure, should accompany changes in CO or arterial compliance, but our results revealed no changes in CO or in arterial compliance. These contradictory results can be understood with the following explanation. PPW and HRV measurements have been demonstrated to be accurate techniques, AIX and PTT are well defined, and their calculations are straightforward with minimal uncertainty [16, 26]. However, similar to other impedance-based bio-signal analyses [27], CO estimation is thought to have limited accuracy, and a subtle change that might have accompanied the acupuncture stimulation may not be traceable using the contemporary impedance cardiography technique [28]. Therefore, contrary to the conclusions of a previous, closely related work [24], we suggest that the increased blood flow velocity, pulse pressure, SEVR, and other hemodynamic variables indicating activated systemic circulation are possibly consequences of the elevated CO, especially in the systolic period, which was induced by the elevated sympathetic tone caused by acupuncture stimulation at ST36. The remaining variables that are not included in the above are provided as the Additional file 1: Figure S2

The most valuable feature of this study is that we simultaneously analyzed various hemodynamic variables during the acupuncture session. Previous studies assessed only one or two variables and could not draw comprehensive conclusions regarding the hemodynamic changes with acupuncture stimulation [8–10]. In this study, however, the simultaneous evaluation of diverse variables related to RPPW, PPG, peripheral arterial blood flow, autonomic nerve activity and cardiac function enabled us to generate an integrated picture of the hemodynamic changes that accompany acupuncture stimulation. Second, we included healthy young adults and strictly followed the predefined inclusion and exclusion criteria to reduce any confounding factors that might be related to the observed hemodynamic changes. On the day of study participation, excessive physical activity and the use of alcoholic or carbonated beverages were prohibited to control for possible influences on the bio-signals. The acupuncture stimulation and the assessment of hemodynamic variables were conducted in an undisturbed room, where only the individual participant and research staff were present. We used a unified data acquisition system, which enabled the simultaneous processing of several hemodynamic variables. These efforts would have contributed to the internal validity of our study. Third, we tried to adopt a similar experimental design and acupuncture method, including acupuncture points and the same type of acupuncture needles, to ensure comparability with related studies [9, 20, 24, 29]. Different acupoints or stimulation types of may induce different changes on bio-signals as there are some previous reports [9, 10, 30, 31]. Through this attempt, we could verify the previously reported hemodynamic effects of acupuncture and advance the knowledge in this area.

Our study has several limitations. Changes in the hemodynamic variables were observed for only a limited time around the acupuncture session. There is controversy over the effective duration related to acupuncture practice. Generally, studies on acupuncture treatment consider both short-term and long-term effects [7]. In this study, however, we focused on the short-term effects of acupuncture and observed hemodynamic changes before, during and directly after acupuncture treatment, which was maintained for 20 min. We found that many variables related to the RPPW, HRV and PPG signals showed significant changes at the last measurement session, implying that prolonged effects of acupuncture might be observed with a longer follow-up design. Second, this pilot study focused on the primary outcome variable with a minimal sample size. Many secondary outcome variables showed consistent patterns during the acupuncture stimulation, but the lack of statistically significant differences from the baseline value might be due to insufficient power. Third, for the assessment of RPPW, we read the pulse signal only at the left Guan. In the general practice of TEAM, practitioners use all three adjacent positions, Cun, Guan, and Chi, at both wrists to diagnose the pulse [32]. In this study, we measured many hemodynamic variables at several time points within a limited time schedule; this allowed only a single-point data acquisition for PPW, which may not reflect the current clinical practice of TEAM. Fourth, physicians typically select more than one acupuncture point for acupuncture treatments. To minimize the control parameters, we selected only ST36 at both legs for acupuncture stimulation. A previous study found that acupuncture stimulation at ST36 could change the bio-signals that are regulated by the cardiovascular system, such as baroreflex function and various hemodynamic variables [33, 34]. Thus, we chose only ST36. Even with this experimental evidence, stimulation at a single acupuncture point cannot appropriately reflect real clinical practice. All of these limitations were partially because of the use of repeated measurements with several devices within a limited time duration and the pioneering aspect of this study. Finally, stress is an important factor in the assessment of bio-signals. To avoid stress, all the participants rested for 20 min before the measurements and acupuncture treatments; nonetheless, we could not ensure that all possible confounding factors of stress were appropriately controlled in this study. In future studies, assessing basal stress levels would be necessary to prevent the inclusion of any participants in a generally stressful condition.

From the analysis of this study, several future research directions can be suggested. Several variables showed consistent trends but lacked statistically significant differences, probably due to the low power of this study. Therefore, a clinical study with a sufficiently large sample size is desired. Second, reflecting actual clinical practice, studies adopting acupuncture stimulations at multiple points are also necessary, and point-specific effects need to be investigated. Third, studies on actual patients need to be conducted. Compared to treatments of healthy subjects, appropriate acupuncture treatments on patients will improve body conditions that should be reflected more dramatically in pulse diagnosis and, thus, in PPW and other hemodynamic variables. In addition, studies with patients would allow disease-specific characteristics of pulse diagnosis to be more clearly identified, which will help elucidate the underlying mechanism of pulse diagnosis in diverse cases.

## Conclusions

In conclusion, in an attempt to find scientific evidence and the mechanism underlying pulse diagnosis, we investigated the changes in RPPW and related bio-signals from PPG, ECG, and ultrasonography with a conventional acupuncture treatment in healthy subjects. The results indicated that ST36 acupuncture stimulations at both legs induced increases in the blood flow velocity in the radial artery and peripheral arterioles and induced a relaxation effect; the treatment increased the radial pulse pressure – especially the systolic pulse pressure and the power spectral density in the low-frequency domain (<10 Hz) – and increased the PPG systolic area and blood flow velocity at the radial artery. In addition, the treatment increased the high-frequency HRV and decreased the low-frequency HRV. Through further analysis, we showed that these increased blood flow velocity indicators were possibly the consequence of increased CO, especially during the systolic period. Using this integrative approach, the analysis of radial pulse pressure can be a predictive tool for the changes in hemodynamic variables during an acupuncture session. Studies with patients and diverse acupuncture point stimulations and more direct measurements of CO are needed to confirm our results and generate a deeper understanding of the mechanism of pulse diagnosis in various health conditions.

## Additional file

Additional file 1: Figure S1. The estimated mean profiles of RPPW variables. The last details are identical with Fig. 4. **Figure S2.** The estimated mean profiles hemodynamic (HRV, PPG, and cardiac output) variables. The last details are identical with Fig. 4. **Figure S3.** The estimated mean profiles ultrasonography variables. The last details are identical with Fig. 4. (DOCX 840 kb)

## Abbreviations

AIX: Augmentation index
ASQ: Acupuncture Sensation Questionnaire
BMI: Body mass index
CEQ: Credibility/Expectancy Questionnaire
CI: Confidence interval
CO: Cardiac output
ECG: Electrocardiogram
GPAQ: Global Physical Activity Questionnaire
HF: High-frequency domain
HRV: Heart rate variability
KMD: Korean medicine doctor
LF/HF: Ratio of low to high frequency
LMM: Linear mixed model
MD: Mean difference
nHF: Normalized high-frequency domain
nLF: Normalized low-frequency domain
NN: Normal to normal interval
PDI: Pulse depth index
PPG: Photoplethysmogram
PPI: Pulse power index
PPW: Pressure pulse wave
PTT: Pulse transit time
PTT: Pulse transit time
PVI: Pulse volume index
RPPW: Radial pressure pulse wave
RSP: Respiration
SD: Standard deviation
SE_0-10Hz_: Spectral energy from 0 to 10 Hz
SE_10-30Hz_: Spectral energy from 10 to 30 Hz
SEVR: Subendocardial viability ratio
SIA: Sensations during insertion of the acupuncture needle
SM: Sensations during maintenance of the acupuncture needle
SMA: Sensations during manipulation of the acupuncture needle
SV: Stroke volume
TEAM: Traditional East Asian medicine
