# Supplementary material for: Hemodynamic changes caused by acupuncture in healthy volunteers: a prospective, single-arm exploratory clinical study
Source: BMC Complement Altern Med. 2017 May 22;17:274. doi: 10.1186/s12906-017-1787-z (PMC5440909; doi:10.1186/s12906-017-1787-z)
Supplement: Additional file 1: Figure S1. — The estimated mean profiles of RPPW variables. The last details are identical with Fig. 4. Figure S2. The estimated mean profiles hemodynamic (HRV, PPG, and cardiac output) variables. The last details are identical with Fig. 4. Figure S3. The estimated mean profiles ultrasonography variables. The last details are identical with Fig. 4. (DOCX 840 kb) [file 12906_2017_1787_MOESM1_ESM.docx]

Additional file 1


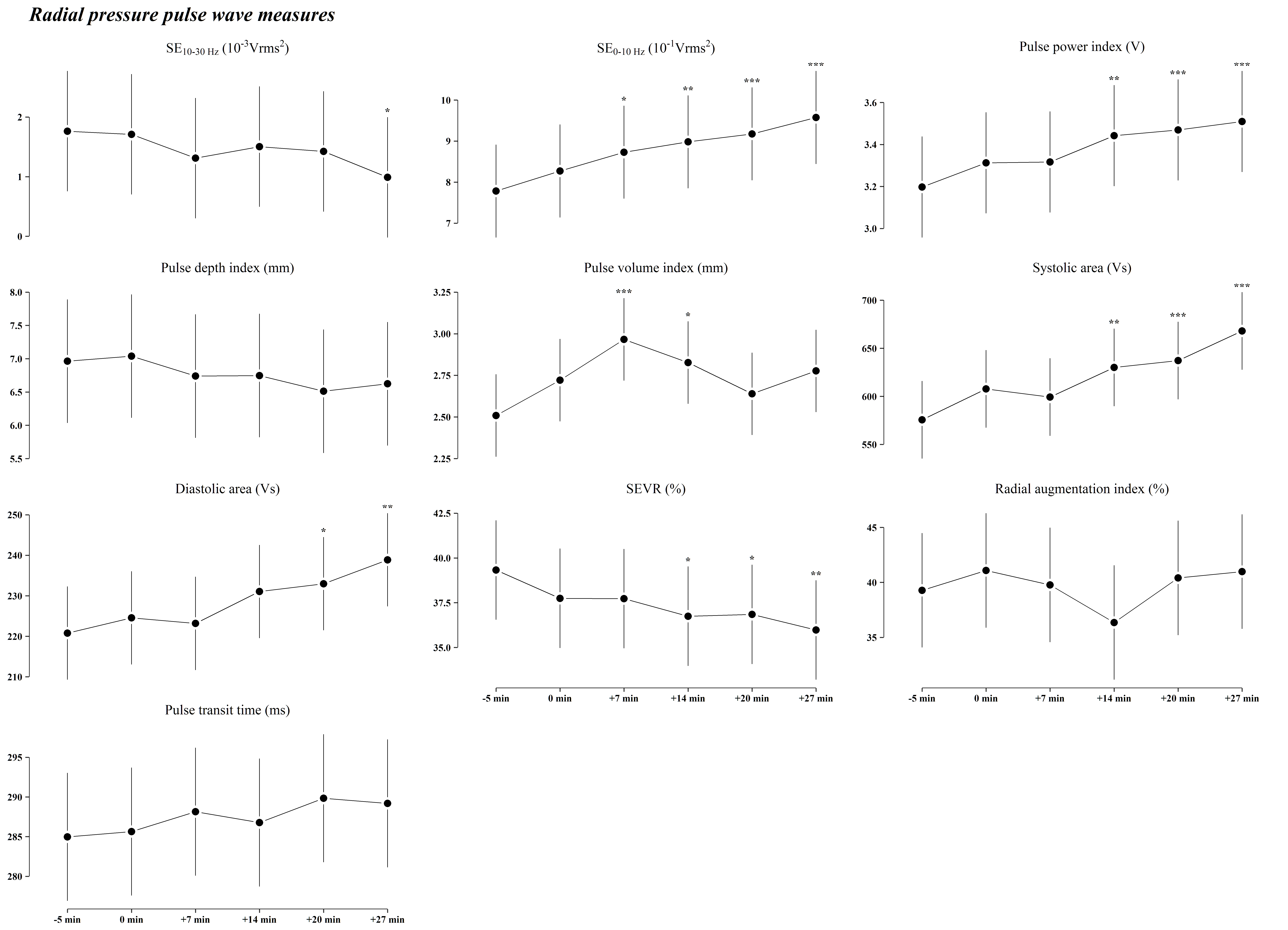


Figure S1. The estimated mean profiles of RPPW variables. The last details are identical with Figure 4.


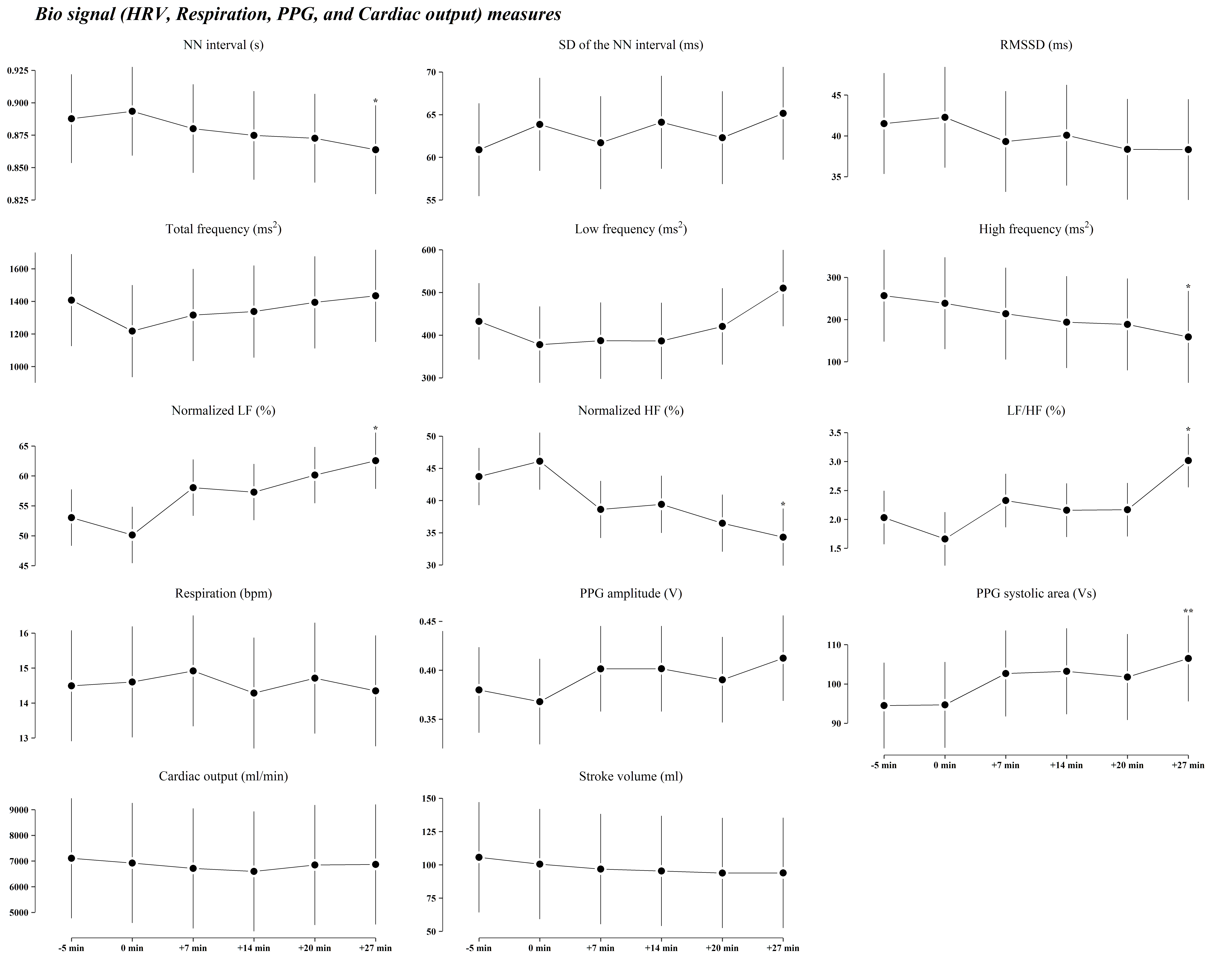


Figure S2. The estimated mean profiles hemodynamic (HRV, PPG, and cardiac output) variables. The last details are identical with Figure 4.


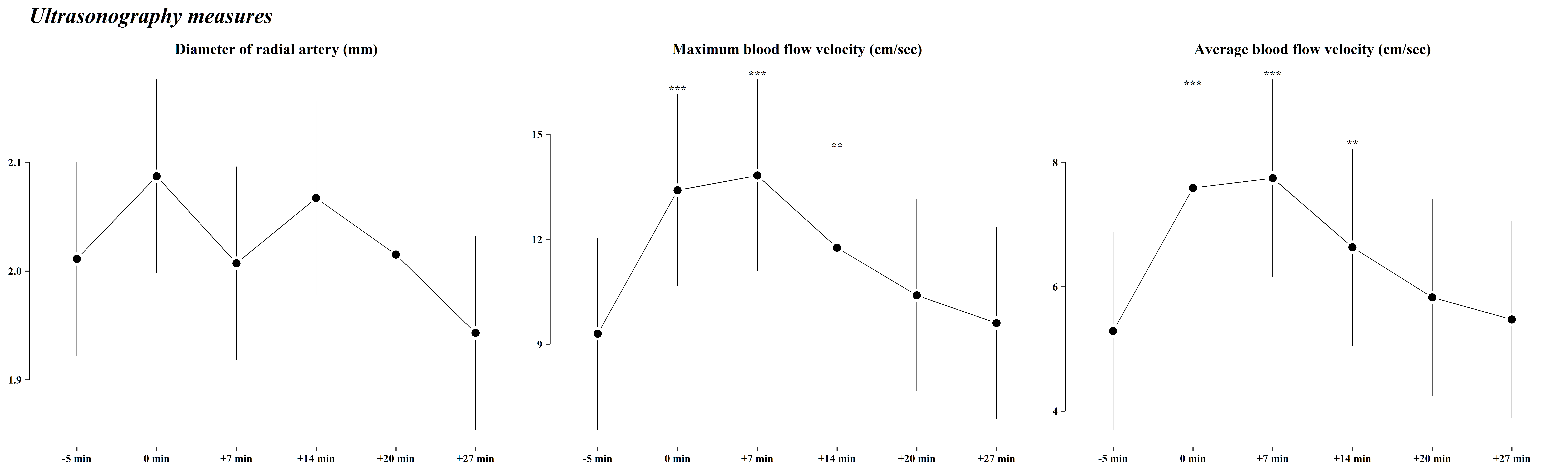


Figure S3. The estimated mean profiles ultrasonography variables. The last details are identical with Figure 4.
